# Imaging findings of human hepatic fascioliasis: a case report and review of the literature

**DOI:** 10.1186/s13256-021-02945-9

**Published:** 2021-06-24

**Authors:** Faeze Salahshour, Abasin Tajmalzai

**Affiliations:** 1 Department of Radiology, Tehran University of Medical Sciences (TUMS), Tehran, Iran; 2 Department of Radiology, Kabul University of Medical Sciences, Kabul, Afghanistan

**Keywords:** Hepatic fascioliasis, Imaging findings, CT, MRI, Case report

## Abstract

**Background:**

Fascioliasis is a food-borne hepatobiliary zoonosis caused by *Fasciola hepatica* and *Fasciola gigantica*. Human infestations are predominantly seen in developing countries where the disease is endemic, but, due to the increase in international travel rates, hepatic fascioliasis is also appearing in nonendemic areas including Europe and the USA. The clinical and laboratory findings are usually nonspecific. Cross-sectional imaging can be very helpful in the diagnosis of fascioliasis as well as to differentiate it from other liver diseases with a very similar clinical picture. The objectives of this case report are to discuss imaging findings of hepatic fascioliasis and to review the literature.

**Case presentation:**

We report the case of a 35-year-old Iranian patient who presented with right upper quadrant pain, low-grade fever, fatigue, and anorexia. The patient had a history of recent travel to the Gilan Province of Iran, almost a month before the onset of symptoms, which is an endemic area of fascioliasis in the country. Laboratory examinations revealed eosinophilia, elevated hepatic enzymes, and slightly raised C-reactive protein. Contrast-enhanced computed tomography of the patient shows clusters of focal ill-defined hypodense lesions with mild peripheral enhancement in the right liver lobe and subcapsular regions. Magnetic resonance imaging of the liver revealed multiple ill-defined lesions of low signal intensity on the T1-weighted image and high signal intensity on the T2-weighted image, extending from the liver capsule into deeper parenchyma toward periportal regions, which shows mild peripheral enhancement on post-contrast images. Imaging-based diagnosis of fascioliasis was made depending on the characteristic distribution of subcapsular tracts/lesions on the above-mentioned imaging, which was then confirmed by serologic tests using enzyme-linked immunosorbent assay. The patient was treated with triclabendazole, showing great clinical improvement, and was eventually discharged in good health condition.

**Conclusion:**

The imaging findings in this case report highlight the importance of cross-sectional imaging for further evaluation of suspected cases of fluke-induced liver disease. The hypothesis of hepatic fascioliasis should be always considered when consistent radiological findings are observed. Clusters of tortuous subcapsular lesions with peripheral contrast enhancement extending into deeper liver parenchyma are characteristic imaging findings that strongly suggest hepatic fascioliasis.

## Background

Fascioliasis is a food-borne hepatobiliary zoonosis caused by *Fasciola hepatica* and *Fasciola gigantica*. Humans ingest infected watercress or contaminated water with encysted larvae (metacercariae), which excyst in the stomach/duodenum, migrate through the intestinal wall to the peritoneal cavity, and then penetrate the Glisson capsule to enter the liver and eventually the bile ducts [3, 4]. The clinical course of fascioliasis consists of two phases: the hepatic parenchymal phase and the ductal phase. The hepatic parenchymal phase usually lasts for 2–4 months, in which immature larvae penetrate the Glisson capsule to invade the liver parenchyma, causing inflammation, abscess formation, hemorrhage, necrosis, granulation, and fibrosis. The ductal phase is characterized by the extension of larvae into the bile ducts where they mature (adult fluke) and start laying eggs that finally pass through the host’s feces. These flukes can survive in the biliary ducts for many years and may cause biliary inflammation and/or obstruction. Infestations in humans are predominantly seen in developing countries including Africa, South America, and North and South Asia (including China and Korea). Due to the increase in international travel rates, hepatic fascioliasis is also appearing in nonendemic areas including Europe and the USA. Misdiagnosis or late diagnosis is still frequently seen because the disease is rarely seen in nonendemic areas and its clinical picture is nonspecific. Clinicians may not be familiar with the presentation, contributing to delayed diagnosis and possible complications that eventually lead to unnecessary surgical interventions. The history of recent travel to the endemic area is very important and should always be considered when consistent radiological findings are observed. Cross-sectional imaging with computed tomography (CT) and/or magnetic resonance imaging (MRI) is necessary when appropriate clinical findings are present [1–4, 8]. The typical imaging findings in this reported case highlight the importance of cross-sectional imaging for further evaluation of suspected cases of fluke-induced liver disease.

## Case presentation

A 35-year-old Iranian patient presented to our hospital with a 4 weeks history of right upper quadrant (RUQ) pain, low-grade fever, fatigue, and anorexia. At admission, her body temperature was 37.8 °C, and her vital signs were within normal limits (blood pressure 122/78 mmHg, pulse rate 75 beats per minute, respiratory rate 14 breaths per minute). On physical examination, mild RUQ tenderness and normal liver span were noticed. No abnormal findings were present on neurological examinations. The patient was a nonsmoker and did not consume alcohol. She was a literate and married woman but had no official duties and was a housewife. She had no history of previous surgery, chronic medical illness, or family/psychosocial history of any health-related conditions including congenital malformation. The patient was receiving no medication before admission to the hospital. The patient had a history of recent travel to the Gilan Province of Iran, almost a month before the onset of symptoms, which is an endemic area of fascioliasis in the country.

**Fig. 1:**
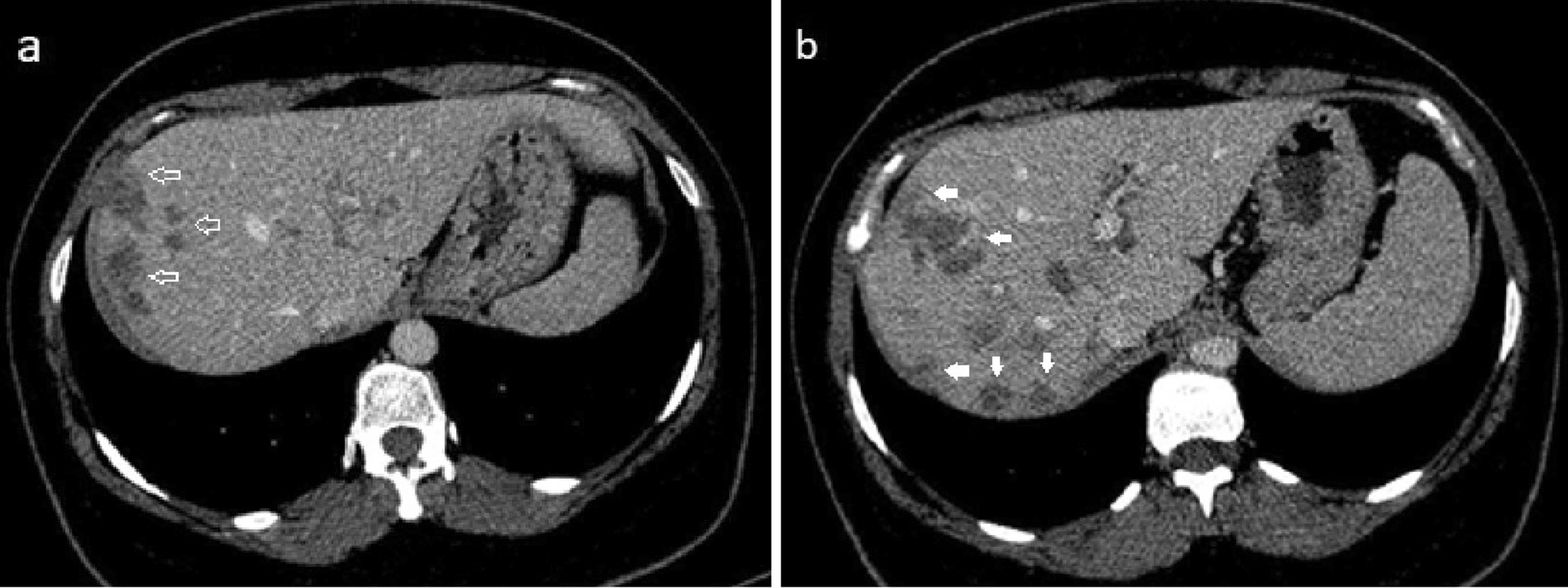
**a** Axial contrast-enhanced CT-image demonstrating patchy areas of decreased attenuation migrating from the liver capsule into deeper parenchyma and periportal regions showing mild peripheral contrast enhancement (arrows). **b** Axial contrast-enhanced CT-image demonstrating clusters of hypodense lesions extending from the liver capsule into deeper parenchyma and periportal regions showing mild peripheral contrast enhancement (arrows)

**Fig. 2:**
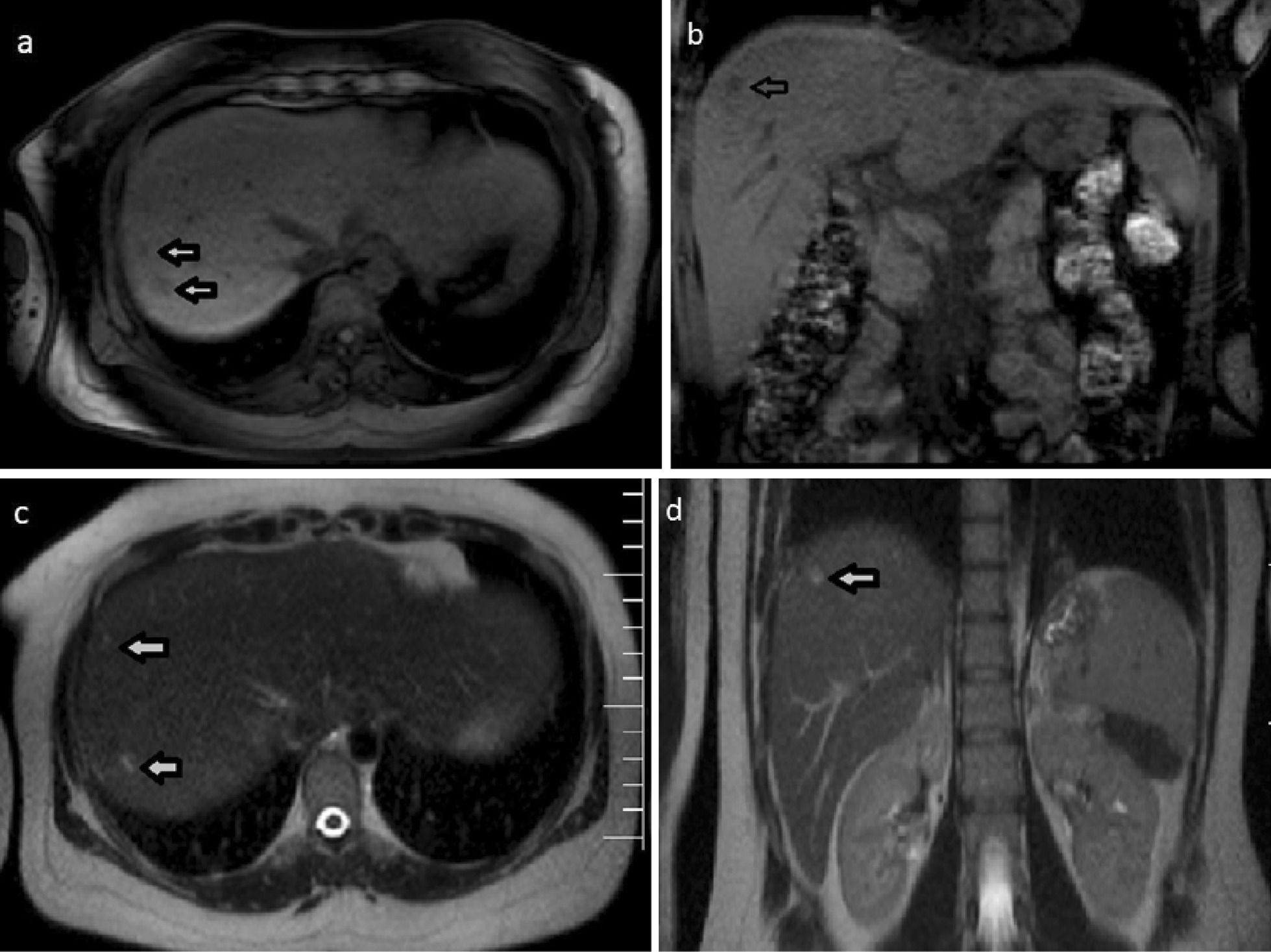
**a** Axial T1W MR image showing hypointense subcapsular lesions at the right lobe of the liver (arrows). **b** Coronal T1W MR image showing a hypointense lesion in the liver (arrow). **c** Axial T2W MR image showing hyperintense subcapsular/parenchymal lesions in the liver (arrows). **d** Coronal T2W MR image showing a hyperintense subcapsular lesion in the liver (arrow)

**Fig. 3:**
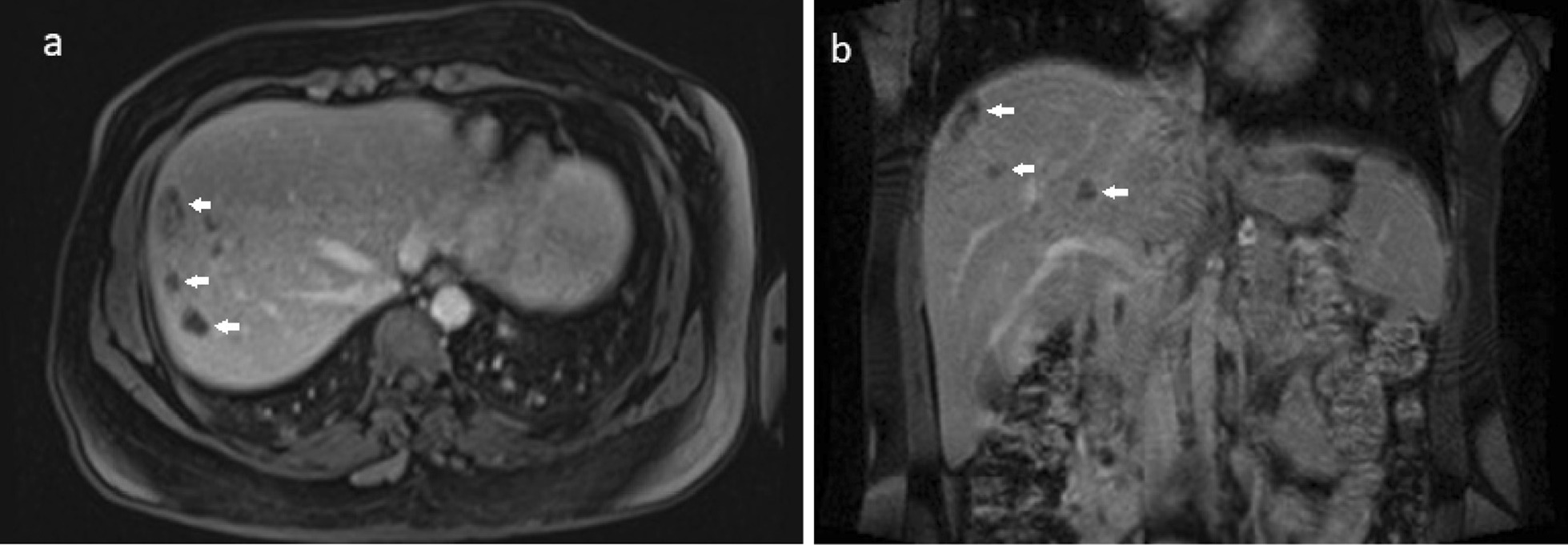
**a** A contrast-enhanced axial T1W MR image showing multiple, round, clustered hypointense subcapsular/parenchymal lesions with mild peripheral contrast enhancement in the liver (arrows). **b** A contrast-enhanced coronal T1W MR image showing multiple, round, clustered hypointense subcapsular/parenchymal lesions with mild peripheral contrast enhancement in the liver (arrows)

A complete blood count (CBC) was performed that revealed a total leukocyte count of 9300/mm^3^ with eosinophilia (eosinophils 15%, neutrophils 61%, lymphocytes 19%, monocytes 5%, and basophils 0%), red blood cell count of 4.2 million/mm^3^, hemoglobin of 12.1 g/dL, and platelet count of 279,000/mm^3^. Laboratory examinations also revealed elevated hepatic enzymes (serum aspartate aminotransferase 76 IU/L, alanine aminotransferase 80 IU/L, lactate dehydrogenase 295 IU/L, alkaline phosphatase 223 IU/L), and slightly raised C-reactive protein 12 mg/L. Renal function tests and serum bilirubin were within normal limits. Abdomen/pelvis CT scan demonstrates clusters of focal ill-defined hypodense lesions in the right liver lobe and subcapsular regions. These clusters of hepatic lesions (abscess) show peripheral contrast enhancement on contrast-enhanced CT (CECT), forming a tunnel-like tract from the entry site at the Glisson capsule deep to the liver parenchyma (tunnels and caves sign) (Fig. 1a, b). No evidence of high-density foci within biliary duct lumen, ductal dilatation, or ductal wall thickening/hyperenhancement was present. Multiplanar dynamic liver MRI was also performed, which shows multiple ill-defined lesions of low signal intensity on T1-weighted image (T1WI) and high signal intensity on T2-weighted image (T2WI) in the right liver lobe, extending from the liver capsule to the deeper parenchyma toward periportal regions (Fig. 2a–d). No bile duct dilatation or obvious low-signal filling defects within the biliary duct lumen were depicted. Contrast-enhanced T1WI shows multiple, round, clustered hypointense lesions with peripheral contrast enhancement in the liver (Fig. 3a, b). These imaging findings were consistent with the hepatic parenchymal phase of the disease in which immature larvae penetrate the liver capsule and invade the liver parenchyma without obvious bile duct involvement. Imaging-based diagnosis of hepatic fascioliasis was made depending on the characteristic distribution of ill-defined subcapsular tracts/lesions with peripheral contrast enhancement extending toward deeper liver parenchyma and periportal regions. The diagnosis was confirmed by serologic tests using enzyme-linked immunosorbent assay (ELISA). For treatment, 500 mg (10 mg/kg) triclabendazole was orally administered in two doses given 12 hours apart. Bed rest, vitamin supplementation, and a protein-rich diet were also recommended. No surgical intervention was needed, and the patient was eventually discharged in good health condition. Sonographic follow-up was advised for the patient at 1-, 3-, and 6-month intervals or until the lesions completely resolved.

## Discussion

In the present study, we report a confirmed case of hepatic fascioliasis in a young woman, presented with nonspecific clinical findings and a recent history of travel to an endemic area of fascioliasis. This case report highlights the importance of cross-sectional imaging in the diagnosis of hepatic fascioliasis as well as to differentiate it from other liver diseases with a very similar clinical and radiological picture. While travel history is most important, characteristic findings of tortuous subcapsular lesions with peripheral contrast enhancement, extending into deeper liver parenchyma on contrast-enhanced CT and MRI strongly suggest hepatic fascioliasis.

In the presence of an appropriate clinical setting, direct parasitological tests, indirect immunological tests, and cross-sectional imaging techniques such as ultrasound, CT, and MRI are used for the diagnosis of hepatobiliary fascioliasis in humans [3]. The diagnostic approach may be variable and depends on some factors, including the degree of suspicion, stage of the disease, and the resources and expertise available [9]. In our case, we have both CT and MRI of the patient, which have a complementary role to each other, and their findings were highly suggestive of hepatic fascioliasis, confirmed by the ELISA test.

In a patient with fascioliasis, common symptoms are right upper quadrant pain, fever, weight loss, fatigue, pruritus, skin rashes, dyspepsia, nausea, and vomiting. Fascioliasis may mimic several hepatobiliary disorders, especially metastatic liver disease. Misdiagnosis or late diagnosis is still frequent and may lead to unnecessary surgical procedures such as cholecystectomy and hepatic segmentectomy [4–6]. Furthermore, delay in treatment may cause the patient to suffer long-standing biliary symptoms and face an increased risk of pigment gallstones. Appropriate imaging is important in differential diagnosis [7].

The best diagnostic clue of hepatic fascioliasis on imaging is clusters of patchy ill-defined hypoenhancing lesions mostly in subcapsular regions that may progress toward deeper parenchyma and periportal areas. Intrahepatic and extrahepatic biliary duct changes are best seen on sonography [8]. Radiological findings of hepatobiliary involvement can be seen as parenchymal/ductal involvement in various imaging modalities as follows: during the parenchymal phase, CT may demonstrate hypodense nodular lesion(s) arising in the subcapsular area in the first weeks after ingestion of the metacercariae, progressing to tortuous clustered lesions by 6 weeks. These clusters of low-density hepatic lesions (abscess) show peripheral contrast enhancement on CECT, forming a tunnel-like tract from the entry site at the Glisson capsule deep to the liver parenchyma (tunnels and caves sign). During the ductal phase, high-density foci within the duct lumen represent trematodes, which are usually associated with ductal dilatation and periportal tracking, ductal wall thickening, and/or hyperenhancement. These ductal phase CT findings are usually seen 10 weeks postinfection. MRI of the liver may demonstrate low-signal T1WI and high-signal T2WI lesions with extension from the liver capsule into the deeper liver. T2WI and magnetic resonance cholangiopancreatography (MRCP) may demonstrate mild ductal dilatation with low-signal filling defects. MRI is better than CT because it can show the characteristic evolutionary pattern of disease that reflects its life cycle in the early parenchymal phase, even without the use of a contrast agent. Also, MRI may provide more details about the course of complications such as hemorrhagic lesions and abscess formation in fascioliasis. MRI can also detect more lesions relative to CT. Sonographic findings of hepatic fascioliasis are usually nonspecific. During the parenchymal phase, they include focal hypoechoic or anechoic liver lesions and diffuse involvement of the liver (heterogeneous echotexture). During the ductal phase, ultrasound typically shows ductal thickening, intrahepatic or common bile duct dilatation, and tortuousness due to the intraluminal parasites or the hemorrhage/inflammatory reaction they incite, and may even show mobile fluke within the dilated bile ducts or gallbladder [1, 3, 8].

Endoscopic retrograde cholangiopancreatography (ERCP) can be performed for both diagnosis and treatment of bile duct obstruction, which allows the detection and extraction of the motile fluke. ERCP findings include linear, elliptical, or rounded filling defects within a dilated biliary ducts or pancreatic duct [8–11].

A summary of the literature regarding imaging findings of human hepatic fascioliasis is presented in Table 1. The findings are almost identical to those in our case.

**Table 1:** Review of the literature of imaging findings associated with hepatic fascioliasis

Studied by	Ultrasound findings	CT findings	MRI findings
Koc *et al*. [1]	Multiple hypoechoic lesions. Heterogeneity of liver parenchyma. Intrahepatic/extrahepatic biliary duct dilatation.	Multiple linear/branching hypodense lesions in subcapsular/peripheral regions. Periportal and peridiaphragmatic lymphadenopathy. Hyperdense foci in the biliary tree.	T2 hyperintense/T1 hypointense nodules with peripheral enhancement. Intrahepatic/extrahepatic biliary dilatation.
Han *et al*. [12]	Hypoechoic tract-like lesions in the liver.	Multiple hypodense lesions forming a tunnel-like tract with peripheral enhancement.	Clusters of T1 isointense and hypointense lesions with peripheral enhancement. Mixed T2 hypointense and hyperintense lesions.
Kaya *et al*. [13]	Not reported	Multiple nodular or tubular branching lesions. Subcapsular hypodensities. Periportal lymphadenopathy.	Not reported
Cantisani *et al*. [14]	Heterogeneity of liver parenchyma. Coalescent nodules in tubular structures. Parasites in the biliary tree.	Nonenhancing hypodense lesions, predominantly in subcapsular regions.	Liver capsule enhancement. Heterogeneity of the liver. Mixed T2 hyperintensity.

Differential diagnosis includes cholangitis and hepatitis, liver abscess, brucellosis, cholecystitis, biliary tract stones, and primary/secondary liver malignancies. Misdiagnosis of fascioliasis is frequent and may lead to unnecessary surgery. The hypothesis of hepatic fascioliasis should be always considered in an appropriate clinical setting when consistent radiological findings are found on cross-sectional imaging [3, 4]. A liver biopsy for tissue diagnosis is rarely performed, which typically reveals necrosis, acute and/or chronic inflammatory changes, debris, and occasionally small fragments of migrating larvae [9]. The final diagnosis should be based on serological tests or visualization of eggs in endoscopically or percutaneously aspirated bile, liver tissue, or stool [7]. Antihelminthic drugs are the primary treatment option. Patients who have a poor response to medications or those with evidence of bile duct obstruction and acute cholangitis may require endoscopic extraction of parasites or decompression/stenting of the biliary system. Gallbladder involvement generally requires cholecystectomy [8].

The following ways can prevent getting infected with hepatic fascioliasis: (1) familiarity of the general public in the endemic areas or those who travel to these areas with this disease and its transmission methods; (2) avoiding consumption of raw wild aquatic plants, local vegetables, raw liver, freshwater fish, or shrimp from areas where *Clonorchis* and *Opisthorchis* infections occur, and give them enough heat before consumption; (3) washing vegetables, especially in endemic regions, with acid detergents and a solution of potassium permanganate [4, 9, 15, 16].

## Conclusion

In general, the diagnosis of *Fasciola hepatica* is particularly challenging in nonendemic areas, as clinical and laboratory findings are usually nonspecific. Cross-sectional imaging is usually performed to look for possible causes of these nonspecific findings. Clusters of tortuous subcapsular tracts/lesions with peripheral contrast enhancement extending into deeper parenchyma and periportal regions are characteristic findings on CT/MRI that strongly suggest hepatic fascioliasis. The hypothesis of hepatic fascioliasis should be always considered in an appropriate clinical setting when consistent radiological findings are observed.

## Data Availability

Supporting data are available, and the corresponding author has the responsibility of access to the data.

## Abbreviations

CBC: Complete blood count
CT: Computed tomography
CECT: Contrast-enhanced computed tomography
ELISA: Enzyme-linked immunosorbent assay
ERCP: Endoscopic retrograde cholangiopancreatography
MRI: Magnetic resonance imaging
MRCP: Magnetic resonance cholangiopancreatography
RUQ: Right upper quadrant
T1WI: T1-weighted image
T2WI: T2-weighted image
